# Demographic buffering in natural populations: A multi‐level perspective

**DOI:** 10.1111/1365-2656.70226

**Published:** 2026-02-03

**Authors:** Gabriel Silva Santos, Samuel J. L. Gascoigne, André Tavares Corrêa Dias, Maja Kajin, Roberto Salguero‐Gómez

**Affiliations:** ^1^ National Institute of the Atlantic Forest (INMA) Santa Teresa Espírito Santo Brazil; ^2^ Department of Ecology, Graduate Program in Ecology and Evolution Rio de Janeiro State University Rio de Janeiro Brazil; ^3^ Department of Biology University of Oxford Oxford UK; ^4^ School of Biological Sciences University of Aberdeen Aberdeen UK; ^5^ Department of Ecology Institute of Biology Universidade Federal do Rio de Janeiro Rio de Janeiro Brazil; ^6^ Department of Biology, Biotechnical Faculty University of Ljubljana Ljubljana Slovenia

**Keywords:** COMADRE Animal Matrix Database, elasticity, environmental stochasticity, life‐history evolution, natural selection, second‐order derivative, sensitivity

## Abstract

1. Environmental stochasticity poses significant challenges to population persistence. A key mechanism thought to buffer populations against such variability is demographic buffering—the ability of a population to stabilise growth despite temporal fluctuations in survival, development or reproduction. However, empirical tests of demographic buffering remain limited and often yield conflicting results. Here, we propose an integrative demographic framework that combines two complementary approaches to identify demographic buffering: (1) stochastic elasticities, which quantify the sensitivity of long‐term stochastic growth rates (*λ*_s_) to variance in demographic processes, and (2) second‐order derivatives of deterministic growth (*λ*₁), which indicate whether selection acts to reduce or amplify variance in vital rates.
2. Applying this framework to 43 natural populations across 37 mammalian species, we position each species along a variance continuum and assess whether those with low stochastic elasticities—suggestive of buffering—also exhibit signs of concave selection on key demographic processes. While most primates and a few other long‐lived mammals occupy the buffered end of the continuum, only one species—the Columbian ground squirrel—exhibits strong support for our hypothesis, with key vital rates both critical for *λ*₁ and under concave selection. In contrast, primates, despite showing low stochastic elasticities, often show convex or absent second‐order effects on their most influential vital rates, indicating a mismatch between ecological buffering and evolutionary constraint.
3. Our findings suggest that demographic buffering is more dynamic and context dependent than previously recognised. Selection does not consistently act to reduce variance in key demographic processes, even in species where population growth appears robust to environmental variability. This decoupling implies that evolutionary and ecological signals of buffering may not always align. Our framework offers a new lens to dissect the demographic and selective processes underpinning resilience, providing a scalable tool for exploring demographic strategies across taxa. Future work integrating phylogenetic context, trait covariation and environmental drivers will be essential to understand the adaptive value of demographic buffering under global change.

## Graphical abstract

We introduce a multi‐level framework that unites stochastic elasticities with nonlinear selection to test demographic buffering. Applying it across mammals reveals a key insight: ecological robustness to variability often decouples from evolutionary constraint, reshaping how we understand resilience under environmental stochasticity.

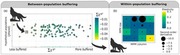

## Resumo

1. A estocasticidade ambiental impõe desafios significativos à persistência populacional. Um mecanismo considerado central para proteger as populações contra tal variabilidade é o tamponamento demográfico (*demographic buffering*)—a capacidade de uma população estabilizar o crescimento apesar das flutuações temporais na sobrevivência, desenvolvimento ou reprodução. No entanto, testes empíricos de tamponamento demográfico permanecem limitados e frequentemente produzem resultados conflitantes. Aqui, propomos uma análise integrativa que combina duas abordagens complementares para identificar o tamponamento demográfico: (1) elasticidades estocásticas, que quantificam a sensibilidade da taxa de crescimento populacional estocástico de longo prazo (*λ*_s_) à variância nos processos demográficos, e (2) derivadas de segunda ordem do crescimento determinístico (*λ*₁), que indicam se a seleção atua para reduzir ou ampliar a variância nas taxas vitais.
2. Aplicando nossa proposta analítica a 43 populações naturais de 37 espécies de mamíferos, posicionamos cada espécie ao longo de um contínuo de variância e avaliamos se aquelas com baixas elasticidades estocásticas—sugestivas de tamponamento—também exibem sinais de seleção côncava em processos demográficos fundamentais. Embora a maioria dos primatas e alguns outros mamíferos de vida longa ocupem a extremidade tamponada do contínuo, apenas uma espécie—o esquilo‐terrestre‐colombiano (*Urocitellus columbianus*)—exibe forte suporte para nossa hipótese, com taxas vitais sendo simultaneamente críticas para *λ*₁ e sob seleção côncava. Em contraste, os primatas, apesar de apresentarem baixas elasticidades estocásticas, frequentemente mostram efeitos de segunda ordem convexos ou ausentes em suas taxas vitais mais influentes, indicando um descompasso entre o tamponamento demográfico e as pressões evolutivas.
3. Nossos resultados sugerem que o tamponamento demográfico é mais dinâmico e dependente do contexto do que reconhecido anteriormente. A seleção não atua consistentemente para reduzir a variância em processos demográficos vitais, mesmo em espécies onde o crescimento populacional parece robusto frente à variabilidade ambiental. Este desacoplamento indica que os sinais evolutivos e ecológicos de tamponamento nem sempre estão alinhados. Nossa abordagem analítica oferece uma nova forma de avaliar os processos demográficos e seletivos que sustentam a resiliência de populações, fornecendo uma ferramenta escalável para explorar estratégias demográficas entre táxons. Trabalhos futuros integrando contexto filogenético, covariação de características e fatores ambientais serão essenciais para compreender o valor adaptativo do tamponamento demográfico sob as mudanças ambientais globais.

## 1 INTRODUCTION

Environmental stochasticity shapes organisms' life histories (Bonsall & Klug, 2011; Stearns, 1992; Tuljapurkar, 1990, 2010). Nonetheless, how organisms will cope with the changing variation in environmental conditions (Bathiany et al., 2018; Boyce et al., 2006; Morris et al., 2008) remains an intriguing ecological and evolutionary question (Sutherland et al., 2013). Evolutionary demography offers a range of explanations for how evolutionary processes influence demographic responses to environmental variability (Charlesworth, 1994; Healy et al., 2019; Hilde et al., 2020; Pfister, 1998; Tuljapurkar et al., 2009). However, it is stochastic demography that explicitly addresses the impacts of fluctuating environments on wild populations of plants and animals (Boyce et al., 2006).

Stochastic demography is grounded in the powerful approximation introduced by Tuljapurkar (1982). This approximation posits that the long‐term stochastic population growth rate (*λ*_*s*_) is directly related to the geometric mean of population growth rates over time (*λ*_*t*_) and the variance–covariance structure of demographic processes (Boyce et al., 2006; Tuljapurkar, 1982). A corollary of this approximation is that increasing temporal variation reduces *λ*_*s*_, thereby diminishing population performance. This phenomenon occurs because *λ*_*s*_ is mathematically defined as the geometric mean of *λ*_*t*_. In turn, increased variation in *λ*_*t*_ leads to a reduction in *λ*_*s*_ (Morris & Doak, 2004; Tuljapurkar, 1982). Consequently, natural selection may favour mechanisms to reduce the temporal variability of key demographic processes (Gaillard & Yoccoz, 2003; Gillespie, 1977; Pfister, 1998).

The ability of a population to diminish the effects of environmental stochasticity on *λ*_*s*_—by reducing the temporal variability of demographic processes that contribute the most to *λ*_*s*_ is called demographic buffering (Gaillard & Yoccoz, 2003; Morris & Doak, 2004; Pfister, 1998). A way to test for demographic buffering is outlined by the demographic buffering hypothesis (Pfister, 1998; Box 1). The demographic buffering hypothesis extends Tuljapurkar's approximation to argue that negative covariance between *the impact of a demographic process on λ*_*t*_ and the extent to *the demographic process varies* over time would enhance the population fitness by buffering against environmental stochasticity, and should therefore be favoured by natural selection (Gaillard & Yoccoz, 2003; Le Coeur et al., 2022; Morris & Doak, 2004; Pélabon et al., 2020; Pfister, 1998). Evidence exists supporting the demographic buffering hypothesis (e.g. Gaillard & Yoccoz, 2003; Rotella et al., 2012) or not (McDonald et al., 2017). However, generalisation of demographic buffering patterns across species remains challenging due to conceptual confusions leading to competing analytical frameworks (Doak et al., 2005; Morris & Doak, 2004).

BOX 1 **Key terminology**

*The demographic buffering hypothesis*: Stemming from Tuljapurkar's approximation (Tuljapurkar, 1982), Pfister (1998) showed that the penalisation term representing the variance–covariance structure tends to be reduced when elasticities of demographic processes and their coefficients of variation covary negatively. However, the term demographic buffering was only coined later (sensu Morris & Doak, 2004). The demographic buffering hypothesis is also referred to as ‘adaptive buffering’ (sensu Le Coeur et al., 2022), suggesting that *selection* acts to minimise the negative impacts of environmental variation by reducing the temporal variance of key demographic processes (e.g. survival, development, reproduction) that have the highest sensitivity/elasticity to population growth rate, a fitness proxy (Gaillard & Yoccoz, 2003; Pfister, 1998).

*Demographic buffering* is a broader concept than the demographic buffering hypothesis; it refers to a population's capacity to withstand environmental variability by keeping essential demographic processes stable over time (Gascoigne, Kajin, & Salguero‐Gómez, 2024; Gascoigne, Kajin, Sepil, & Salguero‐Gómez, 2024; Hilde et al., 2020; Morris & Doak, 2004; Pfister, 1998). It is worth noting that this term does not explicitly allude to the evolutionary mechanisms that include selection, which are predicted by the demographic buffering hypothesis (Le Coeur et al., 2022).

*Demographic lability*: A population's ability to accommodate fluctuations in demographic processes in response to temporal variations in environmental conditions (Jäkäläniemi et al., 2013; Koons et al., 2009; Le Coeur et al., 2022). The relationship between the labile demographic process and the environment can be convex, concave or linear. A labile vital rate in a variable environment will have an average value that is greater than, less than or equal to the vital rate estimated in the mean environment, depending on the shape of the relationship. Similar to the demographic buffering hypothesis, the demographic lability hypothesis relies on *selection* for demographic process to track environmental fluctuations in a way that increases the long‐term fitness (*λ*_*s*_). This process occurs when the increase in demographic process mean—due to convexity—overcomes the detrimental effect of temporal variance in annual population growth rates (Le Coeur et al., 2022).

*Sensitivity*: Represented by a first‐order partial derivative of population growth rate with respect to each demographic process (Caswell, 1978, 2001; Ebert, 1999), sensitivity measures the absolute change in fitness that a change in a demographic process would cause.

*Elasticity*: Proportional sensitivity: A measure of proportional change in fitness caused by a proportional change in demographic process. Elasticities can be of different types (de Kroon et al., 1986, 2000; Grant et al., 2007; Haridas et al., 2009; Haridas & Tuljapurkar, 2005, 2007; Tuljapurkar et al., 2003; Van Tienderen, 2000) and with respect to both the stochastic and the deterministic population growth rates.

*Tuljapurkar's approximation*: To overcome dealing with complex probability distributions that describe demographic fluctuations through time, the approximation captures the effect of temporal variability, at least for small amounts of variability (i.e. small noise) on population growth. It states that the logarithm of the long‐term stochastic population growth rate equals the geometric mean growth rate plus a penalty term containing the demographic process variance–covariance structure (Tuljapurkar, 1982). For our work, we use the derivation of Tuljapukar's approximation from Haridas and Tuljapurkar (2005) where the impact of temporal variability is written in terms of stochastic elasticities of variance—that is, $\log\left(\lambda_{s}\right)\approx \log\left(\lambda_{0}\right)+\left(1/2\right)\times \sum E^{S^{\sigma }}$.

### 1.1 Historical challenges and interpretations of demographic buffering

The correlational approach is the most widely used way to test for demographic buffering, following Pfister (Pfister, 1998; see also Hilde et al., 2020 for a review). In this approach, a negative correlation is expected to emerge by regressing the proportional contribution of vital rates to population fitness (the so‐called elasticity) against its variability (coefficient of variation (CV)). Even for this straightforward approach, two possible interpretations emerge: an evolutionary and a population‐level perspective. In the evolutionary perspective, a significant negative correlation (*ρ* < 0, at a significant level *p* < 0.05) is interpreted as evidence that selection canalises variance in key demographic processes, thus supporting the demographic buffering hypothesis (e.g. Pfister, 1998; Reed et al., 2010; Rotella et al., 2012). The constraint on the temporal variance of influential vital rates forms the basis of a second interpretation focused on the population level (e.g. Li & Ramula, 2015; McDonald et al., 2017). At the population level, the magnitude of *ρ* itself is used to place species along a continuum from more buffered to less buffered. This interpretation assumes that more negative values of *ρ* indicate populations that may be less sensitive to environmental stochasticity through reduced temporal variability in their most important vital rates—as determined by deterministic elasticity values.

Both interpretations face fundamental limitations. A correlation, even if significant, does not demonstrate an underlying mechanism of natural selection. Most importantly, no single population should be expected to exhibit a uniform negative trend in all vital rates. Even the demographic buffering hypothesis does not assume this negative relationship (Hilde et al., 2020); instead, this hypothesis assumes that the vital rates that contribute less to population growth should be under lower selective pressure to reduce their temporal variation, so those vital rates may be ‘free’ to vary through time (Hilde et al., 2020; Morris & Doak, 2004; Reed & Slade, 2012). For this reason, we should not expect a global trend supporting buffering towards a negative correlation nor the magnitude of *ρ* placing populations along a gradient from more to less buffered. Therefore, using *ρ* as a buffering metric is only an indirect predictor at best (and we argue an unlikely one) of the capacity of the populations to reduce the negative impact of the environmental stochasticity.

An alternative to the correlational approach is based on the ‘penalisation term’ ($\sum E^{S^{\sigma }}$) of Tuljapurkar's approximation (Box 1; Tuljapurkar, 1982). This approach quantifies the penalty imposed by the variance of vital rates on the long‐term stochastic population growth rate ($\lambda_{s}$), as shown by the approximation $\log\left(\lambda_{s}\right)\approx \log\left(\lambda_{0}\right)+\left(1/2\right)\times \sum E^{S^{\sigma }}$ where $\lambda_{0}$ represents the population growth rate associated with mean demographic rates (explained in detail by Haridas & Tuljapurkar, 2005 in Equation 5). Lower values of $\sum E^{S^{\sigma }}$, which is always negative, indicates a stronger penalty imposed by variance in vital rates on the long‐term stochastic growth rate ($\lambda_{s}$)—thereby indicating a less buffered population. Reduced variability in vital rates—particularly in those that most affect *λ*_*s*_—leads to $\sum E^{S^{\sigma }}$ value closer to zero ($\sum E^{S^{\sigma }}\approx 0$), reflecting a higher buffering capacity to reduce negative impacts of stochasticity.

Unlike *ρ*, which is an indirect metric for buffering, $\sum E^{S^{\sigma }}$ is directly derived from the roots of the demographic buffering hypothesis—Tuljapurkar's approximation (Box 1; Tuljapurkar, 1982). Importantly, $\sum E^{S^{\sigma }}$ offers a more mechanistic and scalable measure of how temporal variability in vital rates impacts long‐term population growth. Moreover, in $\sum E^{S^{\sigma }}$, the vital rates are not assumed to follow an emergent trend as expected in the correlational approach. However, merging all vital rates into a single value, $\sum E^{S^{\sigma }}$ or *ρ*, hides the direction and strength of natural selection that acts on each vital rate. Crucially, while demographic buffering is a by‐product of the evolutionary process that reduces variance in key vital rates, the penalisation term $\sum E^{S^{\sigma }}$ measures the net ecological outcome of all sources of temporal variability (Haridas & Tuljapurkar, 2005). Thus, $\sum E^{S^{\sigma }}$ is more directly aligned with the general concept of buffering in population dynamics than its evolutionary basis (Miles et al., 2024; Gascoigne, Kajin, & Salguero‐Gómez, 2024). Thus, a weak effect on $\sum E^{S^{\sigma }}$ in *λ*_s_ (i.e. $\sum E^{S^{\sigma }}\approx 0$) is an expected but not sufficient condition for the evidence of the demographic buffering hypothesis.

### 1.2 Towards an integrative framework

So far, correlational and penalisation methods have been used independently in the literature, as they address different aspects of demographic buffering. However, we argue that the different aspects addressed by these approaches must be integrated. The correlational method emphasises the evolutionary bases of demographic buffering (Gaillard & Yoccoz, 2003; Hilde et al., 2020; Pfister, 1998), whereas the penalisation method focuses on population responses to environmental stochasticity (Haridas & Tuljapurkar, 2005; Morris et al., 2008). We use the latter method to compare the demographic buffering patterns between species and identify the populations displaying buffering signatures—meaning $\sum E^{S^{\sigma }}\approx 0$. Therefore, to address the evolutionary prediction that the most important vital rates should be under stronger selective pressure, we propose that, in addition to measuring the $\sum E^{S^{\sigma }}$ for each population, one should also examine the effects of each demographic process within the life cycle on *λ*_*t*_ (e.g. Caswell, 1978, 1996, 2001; de Kroon et al., 1986; Ebert, 1999). This next step aligns with the correlational approach, relating the variance of demographic processes to their influence on *λ*_*t*_. However, because correlation alone cannot confirm causality, it is essential to extend the demographic buffering framework to explore the role of non‐linear selection pressures, which can be assessed through the second‐order derivative (Box 1).

First‐ and second‐order effects of the variation in demographic processes on fitness are evidence of *average* selection pressures over time (Carslake et al., 2008; Caswell, 2001; Kajin et al., 2023; Shyu & Caswell, 2014; Tuljapurkar et al., 2023). The first‐order derivatives of the deterministic population growth rate (*λ*₁), known as *elasticities* (∂*λ*₁/∂*a_ij_*), measure the proportional change in *λ*₁ resulting from a small change in a demographic process (*a*_*ij*_). They describe the linear effect of each vital rate on fitness and can be interpreted as proxies for selection gradients (Caswell, 2001; Lande, 1982). Second‐order derivatives (∂^2^ *λ*₁/∂*a_ij_* ^2^) extend this concept by quantifying how *λ*₁ responds to non‐linear changes in the same demographic process. In other words, they measure the *curvature* of *λ*₁ with respect to each *a*_*ij*_, indicating whether selection acts to amplify or reduce temporal variability in that rate (Caswell, 1996; Shyu & Caswell, 2014).

The sign (>0 or <0) of the second derivatives determines the type of non‐linear selection that acts on the temporal variability of the demographic process. Negative values (concave selection, ∩‐shaped) reduce temporal variance, characteristic of buffering (Caswell, 1996, 2001; Shyu & Caswell, 2014). Positive values (convex selection, ∪‐shaped) indicate selection forces that amplify the temporal variance, revealing a lack of selection pressures on reducing the temporal variance in a demographic process (Caswell, 1996, 2001; Koons et al., 2009; Le Coeur et al., 2022; Shyu & Caswell, 2014). These second derivatives can be further classified as self‐ or cross‐second derivatives, depending on whether they involve one or more than one demographic process. Here, we focus on self‐second derivatives, which quantify the curvilinear impact of an individual demographic process on population growth (i.e. ∂^2^ *λ*₁/∂*a_ij_* ^2^).

Here, we show how the combination of both aforementioned demographic methods—$\sum E^{S^{\sigma }}$ and second derivatives—can be used to test the following hypothesis: buffered species with low summed effect of temporal variability on their fitness should show signatures of concave selection that act to reduce the variance in their most important demographic process(es) (see Box 1 for definitions). Concave selection pressures in a demographic process implicate a reduced sensitivity of population growth to temporal variance in that demographic process (Caswell, 2001; Shyu & Caswell, 2014), thereby enhancing population persistence in the face of environmental stochasticity. We test our hypothesis and demonstrate the applicability and challenges of our framework using 43 populations of 37 mammal species.

## 2 METHODS

### 2.1 Population models—The building blocks to test the demographic buffering hypothesis

Current evidence for demographic buffering has primarily been assessed using matrix population models (*MPMs*, hereafter) (Pfister, 1998; Rotella et al., 2012). However, integral projection models (*IPMs*) (Easterling et al., 2000; Ellner et al., 2016; Gascoigne et al., 2025; Rodríguez‐Caro et al., 2021; Wang et al., 2023) can also identify demographic buffering. MPMs and IPMs are structured, discrete‐time demographic models (Caswell, 2001; Ellner et al., 2016). For simplicity, here we focus on MPMs, but note that the same proposed approach applies to IPMs (Doak et al., 2021; Griffith, 2017). Hereafter, we refer to demographic processes in the MPM ***A*** as its entries *a*_*ij*_ (i.e. upper level parameters sensu Zuidema & Franco, 2001) and the vital rates composing those matrix elements (i.e. lower level parameters, *ditto*). The conversion between matrix elements and vital rates is straightforward (Franco & Silvertown, 2004).

### 2.2 Comparing population into a continuum from less to more buffered—The buffering signature

We used $\sum E^{S^{\sigma }}$ to place species on a variance continuum. The variance continuum represents the *summed* effects of proportional increases in temporal variability across all demographic processes (*a*_*ij*_) of the MPM ***A*** on the population growth rate *λ*_*s*_, operating at the *between‐populations level*. We derived $\sum E^{S^{\sigma }}$ from the summation of elasticities $\sum E^{S}$, which can be partitioned into two components: (i) the sum of stochastic elasticities with respect to variability ($\sum E^{S^{\sigma }}$)—assessing how variability in *a*_*ij*_ affects *λ*_*s*_ and (ii) the sum of stochastic elasticities with respect to the arithmetic mean of demographic processes ($\sum E^{S^{\mu }}$)—assessing the impact of a change in mean values of demographic processes on *λ*_*s*_ (Haridas & Tuljapurkar, 2005). As previously mentioned, a weak $\sum E^{S^{\sigma }}$ (i.e. $\sum E^{S^{\sigma }}\approx 0$) means that the population growth rate is relatively unaffected by the variability in demographic processes (Haridas & Tuljapurkar, 2005), which is consistent with demographic buffering, but needs to be confirmed with a second approach to test for the selective pressures acting in the vital rates. This can be done by using the second derivatives of the population growth rate with respect to demographic processes.

### 2.3 Demographic processes, their first‐ and second‐order effects on *λ*_*s*_ and types of selection on temporal variance

**FIGURE 1:**
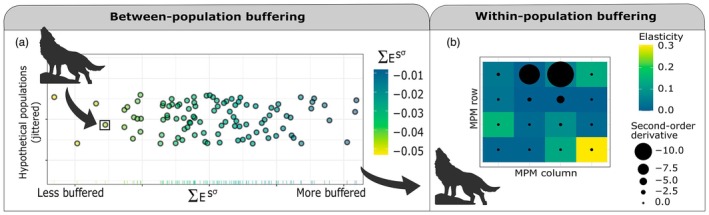
Integrative multilevel framework for assessing demographic buffering. (a) At the between‐population level, populations are positioned according to their summed stochastic elasticities of variance ($\sum E^{S^{\sigma }}$), quantifying the sensitivity of the long‐term growth rate (*λ*_*s*_) to environmental fluctuations. Proximity to zero (right‐hand side) indicates weaker impacts of demographic variance on *λ*_*s*_, characterising potentially *buffered life cycles* (blue dots). Conversely, the left‐hand side represents populations where variability results in strong impacts on *λ*_*s*_ (yellow dots), identifying potentially *unbuffered life cycles*. Data points are jittered along the *y*‐axis to improve visual comprehension. (b) To reveal the within‐population buffering mechanism for a hypothetical wolf population, first‐ and second‐order derivatives are employed. Matrix colours represent first‐order derivatives (the elasticities) of the deterministic growth rate (*λ*_1_), identifying demographic rates with the highest contributions to fitness (yellow cells: High; blue cells: Low). Overlaid black dots denote second‐order derivatives, revealing the selection forces acting on each demographic rate; the co‐occurrence of high elasticity and negative curvature (concave selection) identifies the specific life‐cycle transitions that underpin the buffering potential observed at the population level.

The following method delves into the *within‐population level* by calculating the partial derivatives of *λ*_1_ (obtained by averaging sequential MPMs throughout the study duration, Figure S1) concerning each separate matrix element *a*_*ij*_ of MPM ***A*** (Figure 1b). This step reveals a first‐order effect of the variation of the demographic process on fitness—the elasticities of *λ*_1_ to changes in the demographic processes. Specifically, elasticities correspond to the relative contribution of an individual demographic process (*a*_*ij*_) to the arithmetic mean population growth rate (*λ*_1_). To quantify the curvilinear impact (e.g. concavity as a signature of demographic buffering) of demographic processes on *λ*_1_, we then evaluate a second‐order effect using self‐second derivatives of *λ*_1_ for each *a*_*ij*_ (Figure 1b; Caswell, 1996; Shyu & Caswell, 2014).

We argue that the joint interpretation of first‐ and second‐order effects of variation on *λ*_1_ provides the platform needed to address our prediction of demographically buffered populations displaying concave selection pressures. To address our hypothesis, we:

1. Place populations along a continuum defined by $\sum E^{S^{\sigma }}$ values.
2. Identify the demographic processes with the highest elasticities for each population.
3. Associate the same demographic processes identified in (2) with negative self‐second derivatives, indicating concave selection.

### 2.4 Showcasing demographic buffering in a hypothetical species

We present these steps on a hypothetical wolf population (Figure 1). In this wolf population, individuals who remain in the fourth stage (MPM element *a*_4,4_) have the greatest impact over *λ*_1_, with the highest elasticity value (Figure 1b, yellow square). However, Figure 1b reveals a weak second‐order effect of element *a*_4,4_ on *λ*_1_, thus implying a weak selection pressure to reduce *a*_4,4_ temporal variance. A combination of a strong first‐order and near‐zero second‐order effects on fitness coincides with a strong linear influence of a change in the mean of *a*_4,4_ on *λ*_1_. However, in this example, there is no evidence of concave selection on *a*_4,4_, as we expected based on the positioning of the wolf population on the left (unbuffered) side of the variance continuum (Figure 1a).

We found evidence of concave selection in the fertility of individuals in the second and third stages of the hypothetical wolf species (Figure 1b, MPM elements *a*_1,2_ and *a*_1,3_, respectively, large black dots). Both fertility elements in this wolf population reveal low elasticities (Figure 1b), but highly negative self‐second derivatives. Such a pattern coincides with strong concave selection acting to reduce temporal variance in wolves' second‐ and third‐stage fertilities. These patterns also reveal that temporal autocorrelation in second‐ and third‐stage fertilities affects population fitness. Nonetheless, the absence of concave selection in the fertility of individuals in the fourth stage (Figure 1b, MPM element *a*_1,4_, small black dot) might suggest a pattern consistent with senescence.

Although not our primary goal, we briefly introduce steps to evidence demographic lability. Compelling lability evidence requires sufficient data across environments (over time or space; but see Perret et al., 2024) to construct reaction norms depicting demographic responses to environmental changes (Drake, 2005; Koons et al., 2009). Non‐linear relationships between demographic processes and the environment must be established based on the reaction norms. Demographic processes where an increase in the mean value has a stronger positive impact on population growth rate than the detrimental effect of increased variance need to be identified. The latter condition is only met when the process–environment reaction norms are convex (Drake, 2005; Koons et al., 2009). However, Barraquand and Yoccoz (2013) show that, even with log‐concave reaction norms, environmental variability can positively affect population growth under certain conditions, such as constant survival or density‐dependent growth. Importantly, species may not be purely buffered or labile; some processes may be buffered, labile and insensitive to environmental variability (e.g. Doak et al., 2005). Deciphering these patterns is a primary research interest in the field.

### 2.5 Demographic buffering in mammals: A case study

We examine the performance of our framework and test the hypothesis that species at the buffered end of the variance continuum display highly negative self‐second derivatives for the governing demographic processes. We use 43 MPMs from 37 mammal species (16 species at the within‐populations level). Mammals are of special interest in the context of demographic buffering for two reasons: (1) mammalian life histories have been well studied (Beccari et al., 2024; Bielby et al., 2007; Gillespie, 1977; Jones, 2011; Stearns, 1983) and (2) some of their populations have already been assessed in terms of demographic buffering, particularly for primates (Campos et al., 2017; Morris et al., 2008, 2011; Reed & Slade, 2012; Rotella et al., 2012). Together, the well‐studied life histories and previous information about the occurrence of buffering in mammals allow us to make accurate predictions and validate the performance of our framework.

We used MPMs (Caswell, 2001) from 43 of 139 studies with mammals available in the COMADRE Animal Matrix Database v.3.0.0 (Salguero‐Gómez et al., 2016). These 43 populations encompass 37 species from eight taxonomic orders. We carefully selected these MPMs in our analyses because their models contain values of demographic processes ($a_{\mathrm{ij}}$) for three or more contiguous time periods (median = 8 years), thus allowing us to obtain the stochastic elasticity of each $a_{\mathrm{ij}}$. Although we are aware that, not all possible temporal variation in demographic processes may have been expressed within this period, we assumed three or more transitions are enough to provide sufficient variation for population comparison (Compagnoni et al., 2023). To mitigate bias in variance estimates, we randomly extracted three MPMs from the existing data for each species (Table S1), calculated the mean of these three MPMs and repeated this process 50 times to obtain mean estimates of $\sum E^{S^{\sigma }}$ and their corresponding standard errors. A detailed description of the analysed data and their original sources are detailed in Table S1. Finally, we included MPMs of *Homo sapiens* to cross‐check our estimates of second‐order derivatives, as it is the only mammalian species where these have been calculated (Caswell, 1996). The data for *H. sapiens* were gathered from 26 modern populations (Keyfitz & Flieger, 1990). Because we use only publicly available data, ethical approval was not required.

At the within‐populations level, we used a subset of 16 populations (including *H. sapiens*) whose MPMs were age‐based. We specifically selected these populations because their life cycles can be summarised by two main demographic processes: survival and contribution to the recruitment of new individuals (Caswell, 2001; Ebert, 1999).

To quantify the variance continuum and calculate $\sum E^{S^{\sigma }}$ for between‐population level comparisons, we followed Tuljapurkar et al. (2003) and Haridas and Tuljapurkar (2005). Next, at the within‐population level, we calculated the deterministic elasticities to each demographic process using the *popbio* package (Stubben et al., 2020). The self‐second derivatives were adapted from *demogR* (Jones, 2007) following Caswell (1996) and applied to the mean MPM of each study. All analyses were performed using R version 4.4.1 (R Core Team, 2024).

## 3 RESULTS

**FIGURE 2:**
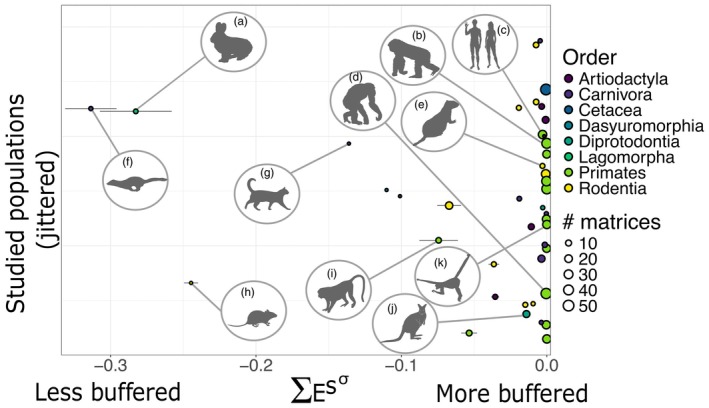
The variance continuum for 43 populations from 37 species of mammals from the COMADRE database based on the summed stochastic elasticities of *λ*_*s*_ to temporal variability in demographic processes ($\sum E^{S^{\sigma }}$) at the between‐populations hierarchical level. Colours represent different taxonomic orders with Primates occupying the right‐hand side. Silhouettes: (a) *Lepus americanus*, (b) *Gorilla beringhei*, (c) *Homo sapiens*, (d) *Pan troglodytes*, (e) *Urocitellus columbianus*, (f) *Mustela erminea*, (g) *Felis catus*, (h) *Rattus fuscipes*, (i) *Erythrocebus patas*, (j) *Macropus eugenii* and (k) *Brachyteles hyphoxantus*. The vertical axis delineates the values of the probability density function, indicating the frequency of populations at each value of $\sum E^{S^{\sigma }}$. The placement of data points (species/populations) along the horizontal axis corresponds to their calculated values of $\sum E^{S^{\sigma }}$ and is arranged linearly, while the placement along the *y*‐axis is random (jittered) for improved visual comprehension.

We ranked 43 populations from 37 mammal species into a continuum of variance according to the summed impact of variation in demographic processes on *λ*_*s*_ (Figure 2). Most of the analysed taxonomic orders were placed on the low or zero variance, end of the variance continuum (Figure 2), coinciding with demographically buffered populations. As expected, all 14 primate populations occupied the buffered side of the variance continuum, with the exception of the Patas monkey (*Erythrocebus patas*, Primates, $\sum E^{S^{\sigma }}$ = −0.0521 ± 5.38 × 10 ^−3^) (Figure 2 silhouette j). The first non‐primate species placed near the buffered end of the continuum was the Columbian ground squirrel (*Urocitellus columbianus*, Rodentia, $\sum E^{S^{\sigma }}$ = −3.38 × 10 ^−3^ ± 6.96 × 10^−4^) (Figure 2 silhouette e). On the other opposite, the species with the highest contribution of variation in demographic processes—placed at the high‐variance end of the continuum—was the stoat (*Mustela erminea*, Carnivora, $\sum E^{S^{\sigma }}$ = −0.310 ± 0.0162) (Figure 2 silhouette f). The snowshoe hare (*Lepus americanus*, Lagomorpha, $\sum E^{S^{\sigma }}$ = −0.262 ± 0.0233) (Figure 2 silhouette a) and the Bush rat (*Rattus fuscipes*, Rodentia, $\sum E^{S^{\sigma }}$ = −0.245 ± 4.29 × 10^−3^) (Figure 2 silhouette h) were positioned on the non‐buffered end of the variance continuum. Additional information (including standard errors of the elasticity estimates) is provided in Table S1.

A posteriori, we quantified the impact of phylogenetic relatedness on the estimates of the sum of stochastic elasticities and then for the correlation between those estimates and the number of MPMs available per species. For the former, we estimated Blomberg's K, a measure of phylogenetic signal that ranges between 0 (weak signal) to positive value 1 (strong) (Münkemüller et al., 2012). Blomberg's *K* in our analyses was 0.23. The correlation between the number of available MPMs per study and the sum of stochastic elasticities (post jack‐knifing) raised a weakly negative coefficient (−0.002), though significant (*p* = 0.017), supporting the robustness of the $\sum E^{S^{\sigma }}$, particularly for further hypothesis testing.

### 3.1 Making sense of the shape of natural selection in the context of demographic buffering

**FIGURE 3:**
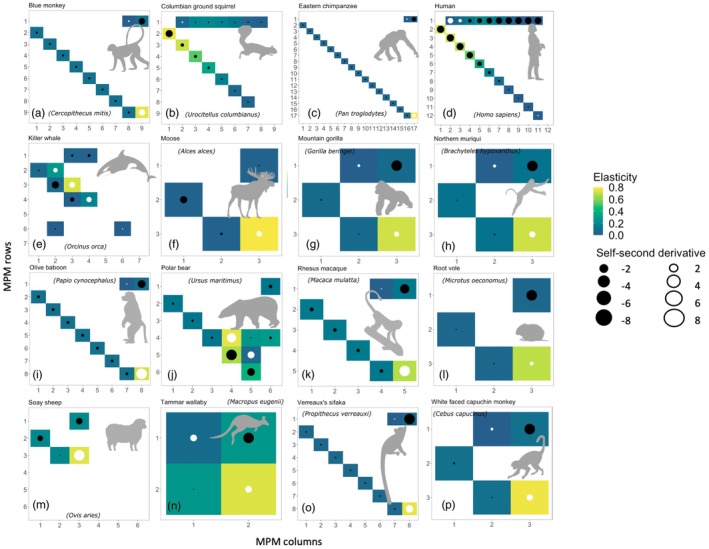
First‐ and second‐order effects on population growth rate, *λ*_1_ (corresponding to elasticities and self‐second derivatives of population growth rate, respectively) for 16 mammal species. The 16 plots represent populations (labels a to p) where the MPMs built by ages were available in the COMADRE Animal Matrix Database. The yellow–blue colour scale represents elasticity values for each of the demographic processes in the MPM, where yellow cells represent high and blue cells represent low elasticity of deterministic population growth rate to changes in demographic processes. No colour means elasticity = 0. The black dots represent negative self‐second derivatives of *λ*_1_—corresponding to concave selection—and the white dots represent positive self‐second derivatives of *λ*_1_—ditto convex selection. The dot sizes are scaled by the absolute value of self‐second derivatives, where the smaller the dot, the closer a self‐second derivative is to 0, indicating weak or no nonlinearity. Thus, large dots indicate strong nonlinear selection forces, either concave (black) or convex (white). Since the derivatives of population growth rate are confounded by eigen structure (de Kroon et al., 2000), the scaling of the elasticity values and second‐derivative values is species specific—that is, each plot has its own scale. Species‐specific scales can be found in the Supporting Information (Table S2).

We found support for our hypothesis—that populations with low $\sum E^{S^{\sigma }}$ should exhibit signs of concave selection reducing variance in most important demographic processes—in only one species: the Columbian ground squirrel (*Urocitellus columbianus*; Figure 2, silhouette e). The strongly negative self‐second derivative with respect to growth from the first to second stage (Figure 3b, MPM element *a*_2,1_) indicates that *a*_2,1_ is both important, and at the same time, kept constant through time in this population of *U. columbianus*.

In humans, the support for our hypothesis was present, but weaker, as humans are placed further away from the buffered end of the variance continuum (Figure 2, silhouette j). However, the demographic parameters representing growth from the first to second age class and growth from the second to third age class (matrix elements *a*_2,1_ and *a*_3,2_, respectively) displayed high elasticities alongside negative self‐second derivatives (Figure 3d), corroborating with the demographically buffered population.

For the remaining studied species, the demographic processes with the highest elasticity values did not display strong negative self‐second derivatives (Figure 3). Particularly for the majority of primates, placed on the buffered end of the variance continuum, demographic processes with high elasticities had positive values for the self‐second derivatives (indicated by yellow squares with white dots in Figure 3). Examples of primate species exhibiting high elasticities and positive values for their self‐second derivatives include northern muriqui (*Brachyteles hypoxanthus*), mountain gorilla (*Gorilla beringei*), white‐faced capuchin monkey (*Cebus capucinus*), rhesus monkey (*Macaca mulatta*), blue monkey (*Cercopithecus mitis*), Verreaux's sifaka (*Propithecus verreauxi*) and olive baboon (*Papio cynocephalus*) (Figure 3). This implies that the key demographic processes influencing *λ*_1_ do not show evidence of selective pressure for reducing their variability.

We also found support for increasing variability of the most important vital rates for populations in the demographic buffering end. For instance, for the Soay sheep (*Ovis aries*), remaining in the third age class (Figure 3m, *a*_3,3_) impacts *λ*_*t*_ the most and is under selection pressure to have its variance increased. These characteristics suggest potential conditions for lability, despite the species being positioned closer to the buffered end of the variance continuum.

Adding the second‐order effect of variation on fitness to the toolbox for demographic buffering is an important addition. The high absolute values of self‐second derivatives (large dots, either black or white, Figure 3) suggest that *λ*_*t*_ is sensitive to autocorrelation in those demographic processes. This pattern also means that if, for example, the mean value of *a*_5,4_ for *Ursus maritimus* increased, the sensitivity of *λ*_*t*_ to *a*_5,4_ would decrease because the self‐second derivative of *a*_5,4_ is highly negative (depicted by the largest black dot in polar bear, Figure 3j). The opposite holds for the *a*_4,4_, where an increase in the value of *a*_4,4_ would increase the sensitivity of *λ*_*t*_ to *a*_4,4_ because the self‐second derivative of *a*_4,4_ is highly positive (the largest white dot in the polar bear MPM).

## 4 DISCUSSION

We explore demographic buffering patterns through the integration of established demographic techniques. Our framework merges insights from both stochastic and deterministic demographic approaches, which revealed only limited support for our hypothesis. Specifically, we had anticipated that species exhibiting minimal influence from temporal variability in demographic processes on their stochastic growth rates would demonstrate concave selection affecting the demographic processes with the highest deterministic elasticities. However, using stochastic elasticities alongside the first‐ and second‐order perturbation analysis of the deterministic population growth rate and applying these analyses to mammal species, we found that only the Columbian ground squirrel fully supported our hypothesis; humans showed partial support; other species did not.

Evidencing demographic buffering is not straightforward. Indeed, through the analysis of stochastic population growth rate (*λ*_*s*_) in our application of the framework to 43 populations of 37 mammal species, we identify the highest density of natural populations near the buffered end of the variance continuum ($\sum E^{S^{\sigma }}$ closer to 0). However, we show that most of the species then fail to exhibit signs of concave (∩‐shaped) selection on impacting demographic parameters, opposed to our hypothesis. Such results suggest discordance between two features of demographic buffering, namely: (1) the stochastic population growth rate having a low sensitivity to temporal variability in demographic processes and (2) demographic processes having their temporal variability reduced by selection.

The lack of association between the non‐linear selection patterns (concave/convex) and species positioning on the variance continuum for the studied mammal species may have several explanations. First, non‐linear selection on demographic process variability is *dynamic* (Kajin et al., 2023). Within a life cycle, even minor changes in key demographic processes can trigger a domino effect, affecting not only the process itself but also the sensitivity of *λ*_1_ to changes in said process (Stearns, 1992). Consequently, correlations between demographic processes (negative correlations known as trade‐offs) are influenced by minor alterations in the governing demographic processes (Doak et al., 2005). Because of these characteristics, second‐order derivatives reveal ‘fine scale’ fitness behaviour compared to sums of stochastic elasticities. Evolutionary demography still requires new tools to connect second‐order fitness effects with stochastic elasticities in a biologically interpretable manner as in Tuljapurkar et al. (2023).

The stochastic elasticities explicitly account for the demographic process variation in time, while the first‐ and second‐order effects on fitness are obtained from temporally averaged population matrices. Because a mean environment rarely characterises the natural variation in demographic process typical of stochastic environments (Boyce et al., 2006), any metric derived from averaged matrix population models represents only an averaged realisation and could only rarely be representative of a pattern emerged from explicitly accounting for temporal variation.

Our original assumptions regarding demographically buffered populations, however, remain valid. We assumed that (1) a buffered population is one with a weak summed effect of temporal variability on the long‐term stochastic population growth rate, $\sum E^{S^{\sigma }}$, and (2) if a population is buffered, there should be signs of concave selection acting on the demographic process with the highest deterministic elasticity. The lack of support for our hypothesis supports the idea that the patterns of first‐ and second‐order effects of demographic process variation on fitness are dynamic and can change rapidly in natural environments. Even if a given demographic process is primarily governing the population growth rate in 1 year, a different one might take over next year (Evers et al., 2021).

When placing our study species along a variance continuum, primates tend to be located on the buffered end. However, most primates displayed convex—instead of the expected concave—selection on adult survival. Similar results, where the key demographic process failed to display reduced temporal variability, have been reported for long‐lived seabirds (Doherty et al., 2004). One explanation for the unexpected convex selection on adult survival involves trade‐offs, as suggested by Doak et al. (2005). When two demographic parameters are negatively correlated, the variance of population growth rate can be increased or decreased (Compagnoni et al., 2016; Evans & Holsinger, 2012).

Correlations among demographic processes (positive and negative) inherently influence the biological limits of variance (Haridas & Tuljapurkar, 2005). This is because the magnitude of variation in a particular demographic process is constrained by the variation of other demographic processes. Not surprisingly, correlations among demographic processes have been shown to be strongly subjected to ecological factors (Fay et al., 2022). Therefore, future studies may benefit from deeper insights using *cross*‐second derivatives (Caswell, 1996, 2001) to investigate correlations among demographic processes.

Biological variance estimates are inevitably subjected to several sources of bias (Simmonds & Jones, 2024). To minimise bias, we randomly sampled the available matrices before obtaining the estimates. Despite the significant correlation between $\sum E^{S^{\sigma }}$ and the number of available matrices per species, the relative positioning of species remains meaningful for between‐population level comparisons, as the correlation is very weak (−0.002). Still, researchers carrying out macroecological comparisons of demographic buffering might want to be even more stringent than we have been here with their data sets, as these grow longer with time (Compagnoni et al., 2021; Salguero‐Gómez, 2021).

Regarding phylogenetic effects, our tests revealed a mild signal, but we note that future work regressing $\sum E^{S^{\sigma }}$ values against potential independent variables (e.g. climate values) may want to correct for this phylogenetic dependence. By having carefully chosen studies from a database that contains >400 species and retained only those that passed through a set of selection criteria (Che‐Castaldo et al., 2020; Gascoigne, Rolph, et al., 2023; Kendall et al., 2019; Römer et al., 2024; Simmonds & Jones, 2024), we mitigate those biases a priori. Furthermore, we are using an elasticity‐based approach, meaning we are comparing proportional variances. At present, the available methods still do not account for constraints in variance nor performing a perturbation approach disproportionately.

The analyses at both between‐ and within‐populations levels are fundamentally interconnected. This connection is grounded on the fact that large, summed elasticities to variability are intrinsically linked to high elasticity values, as demonstrated in Equation 6 (Haridas & Tuljapurkar, 2005). This finding robustly endorses the perspective that species' positions along the variance continuum should be interpreted with consideration of first‐ and second‐order effects, and additionally, in the context of selection pressures acting on the variability of demographic processes, as revealed by second‐order derivatives.

Demographic processes within our study populations often face a mix of convex and concave selection. This mix of selection patterns was suggested by Doak et al. (2005), who noted that dramatic changes in population growth rate sensitivities are influenced by correlations among demographic processes. Here, only two of the 16 mammal species revealed concave selection on the key demographic processes: Columbian ground squirrel (*Urocitellus columbianus*) and humans (*Homo sapiens*). These two species were placed near (or relatively near) the buffered end of the variance continuum, supporting (partially) our hypothesis. Evidence of buffering has been reported across 22 ungulate species (Gaillard & Yoccoz, 2003). However, in the one ungulate we examined, the moose (*Alces alces*), we found only partial support for our hypothesis, as it is near the buffered end of the variance continuum but lacks concave selection pressures on the most important demographic process.

Our overall findings reveal varying levels of support for the notion that adult survival in long‐lived species tends to be buffered. Indeed, Gaillard et al. (1998) found that adult female survival varied considerably less than juvenile survival in large herbivores. This finding was also supported by further studies in ungulates (Gaillard & Yoccoz, 2003), turtles (Heppell, 1998), vertebrates and plants (Pfister, 1998). Gaillard and Yoccoz (2003) reported unexpectedly high adult survival in small mammals, even though the studied small mammals were annual, and as such, comparable to a large mammal model. Seasonality, frequency and method of sampling all influence survival estimates and their estimated variability; thus, when comparing multiple species/studies, all the latter characteristics should be taken into account when interpreting the results.

Although species at the buffered end of the variance continuum tend to be slow‐living mammals, this pattern is not simply a reflection of the slow–fast life‐history continuum. The fast–slow axis captures evolutionary pacing of survival and reproduction (Stearns, 1976), whereas the buffering continuum quantifies the demographic sensitivity of *λ_s_* to temporal variance in vital rates (Haridas & Tuljapurkar, 2005; Stott et al., 2024). Once environmental stochasticity and phylogenetic relatedness are accounted for, the explanatory power of life‐history pace reduces (e.g. Santos et al., 2024). Thus, both continua are related but operate at distinct scales—evolutionary versus demographic.

Examining the drivers of demographic buffering has become an important piece of the ecological and evolutionary puzzle of demography. As such, understanding buffering can help us better predict population responses to environmental variability, climate change and direct anthropogenic disturbances (Boyce et al., 2006; Gascoigne, Kajin, & Salguero‐Gómez, 2024; McDonald et al., 2017; Pfister, 1998; Salguero‐Gómez, 2024; Vázquez et al., 2017). By setting demographic buffering into a broader and more integrated framework, we hope to enhance comprehension and prediction of the implications of heightened environmental stochasticity on the evolution of life‐history traits. This understanding is crucial in mitigating the risk of extinction for the most vulnerable species.

## AUTHOR CONTRIBUTIONS

Gabriel Silva Santos developed the initial concept, performed the statistical analyses and contributed to the first draft of the manuscript. Samuel J. L. Gascoigne developed the initial concept, contributed to the first draft and all other versions of the manuscript and generated final figures. André Tavares Corrêa Dias co‐advised the project and contributed significantly to final versions of the manuscript. Maja Kajin developed and managed the project, contributed to the first draft and all other versions of the manuscript and generated final figures. Roberto Salguero‐Gómez developed and managed the project and contributed to the first draft and all other versions of the manuscript. All authors made substantial contributions in editing the manuscript and further refining ideas and interpretations.

## CONFLICT OF INTEREST STATEMENT

The authors declare no conflicts of interest.

## Supporting information


**Table S1.** The metadata used and the respective results presented in the main text.
**Figure S1.** Histogram showing the distribution of sampling effort duration (in years) across the 43 studied mammal populations. The *x*‐axis represents the duration of the study (“Years sampled”).
**Table S2.** The species‐specific scales for the elasticity of *λ*_1_ to changes in demographic processes and for the self‐second derivatives of *λ*_1_ with respect to demographic processes for the 16 mammal species studied.

## Data Availability

The demographic data used in this paper are open access and available in the COMADRE Animal Matrix Database (https://compadre‐db.org/Data/Comadre). A list of the studies and species used here is available in the Supporting Information (Table S1). The data and code supporting the results are available at Zenodo (https://zenodo.org/records/18300981).
