# Supplementary material for: Demographic buffering in natural populations: A multi‐level perspective
Source: J Anim Ecol. 2026 Feb 3;95(8):1298–310. doi: 10.1111/1365-2656.70226 (PMC13439591; doi:10.1111/1365-2656.70226)
Supplement: Supplementary file 1 [file JANE-95-1298-s001.docx]

**Supplementary material for**

**Demographic buffering in natural populations: A multi-level perspective**

**Table S1.** The metadata used and the respective results presented in the main text. The first three columns represent the source information for the Matrix Population Models (MPMs), extracted precisely as presented in COMADRE 3.0.0. We inform the total number of available MPMs in the COMADRE database, the deterministic (λ_1_) and stochastic (λ_s_) population growth rate, as well as the sum of stochastic elasticities with respect to variability ${\Sigma E}_{a_{ij}}^{S}$ and its standard error ${\Sigma E}_{a_{ij}}^{S}(SE)$. Note that ${\Sigma E}_{a_{ij}}^{S}$ and its standard error are informed in scientific notation due to their small order of magnitude.

| **Species** | **Common name** | **Order** | **Number of available MPMs** | $\lambda_{1}$ | $\lambda_{s}$ | $\Sigma\boldsymbol{E}_{\boldsymbol{a}_{\boldsymbol{ij}}}^{\boldsymbol{S}^{\boldsymbol{\sigma}}}$ | $\Sigma\boldsymbol{E}_{\boldsymbol{a}_{\boldsymbol{ij}}}^{\boldsymbol{S}^{\boldsymbol{\sigma}}}$**(SE)** |
| --- | --- | --- | --- | --- | --- | --- | --- |
| *Alces alces* | Moose | Artiodactyla | 14 | 1.21 | 1.21 | -6.69E-04 | 8.42E-05 |
| *Antechinus agilis* | Agile antechinus | Dasyuromorphia | 3 | 0.93 | 0.89 | -1.11E-01 | 1.62E-03 |
| *Bos primigenius* | Cattle | Artiodactyla | 8 | 1.00 | 1.00 | -2.83E-03 | 2.96E-04 |
| *Brachyteles hypoxanthus* | Northern muriqui | Primates | 25 | 1.05 | 1.05 | -5.31E-05 | 2.09E-05 |
| *Brachyteles hypoxanthus* | Northern muriqui | Primates | 25 | 1.11 | 1.11 | 1.22E-05 | 5.05E-06 |
| *Callospermophilus lateralis* | Golden-mantled ground squirrel | Rodentia | 18 | 2.05 | 1.97 | -6.68E-02 | 8.72E-03 |
| *Cebus capucinus* | White faced capuchin monkey | Primates | 22 | 1.02 | 1.02 | -2.04E-04 | 4.75E-05 |
| *Cebus capucinus* | White-faced capuchin monkey | Primates | 22 | 1.06 | 1.06 | -1.03E-04 | 2.85E-05 |
| *Cercopithecus mitis* | Blue monkey | Primates | 28 | 1.04 | 1.04 | -4.43E-05 | 1.18E-05 |
| *Cervus canadensis subsp. nelsoni* | Rocky Mountain elk | Artiodactyla | 10 | 1.11 | 1.10 | -8.55E-03 | 1.09E-03 |
| *Chlorocebus aethiops* | Vervet | Primates | 8 | 1.19 | 1.15 | -8.03E-02 | 1.31E-02 |
| *Erythrocebus patas* | Patas monkey | Primates | 9 | 1.13 | 1.09 | -5.21E-02 | 5.38E-03 |
| *Eumetopias jubatus* | Northern sea lion; Steller sea lion | Carnivora | 4 | 0.90 | 0.90 | -4.52E-03 | 2.44E-04 |
| *Felis catus* | Feral cat | Carnivora | 3 | 1.95 | 1.83 | -1.34E-01 | 1.89E-03 |
| *Gorilla beringei* | Mountain gorilla | Primates | 41 | 1.03 | 1.03 | -1.28E-05 | 1.32E-05 |
| *Gorilla beringei subsp. beringei* | Mountain gorilla | Primates | 41 | 1.05 | 1.05 | -6.81E-05 | 1.11E-05 |
| *Hippocamelus bisulcus* | Huemul deer | Artiodactyla | 3 | 1.00 | 1.00 | -1.80E-03 | 1.09E-04 |
| *Homo sapiens sapiens* | Human | Primates | 26 | 1.06 | 1.06 | -2.24E-03 | 3.15E-04 |
| *Leopardus pardalis* | Ocelot | Carnivora | 4 | 1.09 | 1.09 | -2.94E-04 | 3.89E-05 |
| *Lepus americanus* | Snowshoe hare | Lagomorpha | 5 | 0.81 | 0.71 | -2.62E-01 | 2.33E-02 |
| *Lycaon pictus* | African wild dog | Carnivora | 3 | 1.50 | 1.43 | -9.70E-02 | 9.91E-04 |
| *Macaca mulatta* | Rhesus macaque | Primates | 24 | 1.13 | 1.13 | -3.84E-04 | 6.83E-05 |
| *Macropus eugenii* | Tammar wallaby | Diprotodontia | 15 | 0.98 | 0.97 | -1.43E-02 | 1.62E-03 |
| *Marmota flaviventris* | Yellow-bellied marmot | Rodentia | 8 | 0.89 | 0.89 | -8.80E-03 | 6.98E-04 |
| *Marmota flaviventris* | Yellow-bellied marmot | Rodentia | 8 | 0.92 | 0.92 | -7.00E-03 | 7.04E-04 |
| *Microtus oeconomus* | Root vole | Rodentia | 28 | 1.03 | 1.03 | -5.60E-04 | 1.06E-04 |
| *Mustela erminea* | Stoat | Carnivora | 4 | 1.26 | 1.07 | -3.10E-01 | 1.62E-02 |
| *Orcinus orca* | Killer whale | Cetacea | 50 | 1.00 | 1.00 | -4.72E-04 | 1.53E-04 |
| *Ovis aries* | Soay sheep | Artiodactyla | 6 | 1.10 | 1.08 | -3.45E-02 | 2.96E-03 |
| *Pan troglodytes subsp. schweinfurthii* | Eastern chimpanzee | Primates | 45 | 0.98 | 0.98 | -1.94E-04 | 5.06E-05 |
| *Papio cynocephalus* | Olive baboon | Primates | 37 | 1.05 | 1.05 | -2.41E-04 | 6.97E-05 |
| *Peromyscus maniculatus* | Deer mouse | Rodentia | 4 | 1.11 | 1.10 | -9.41E-03 | 6.88E-04 |
| *Phascolarctos cinereus* | Koala | Diprotodontia | 4 | 1.06 | 1.06 | -2.53E-03 | 2.16E-04 |
| *Phocarctos hookeri* | New Zealand sea lion | Carnivora | 16 | 1.02 | 1.02 | -3.56E-03 | 4.15E-04 |
| *Propithecus verreauxi* | Verreaux's sifaka | Primates | 24 | 0.99 | 0.99 | -3.06E-04 | 6.29E-05 |
| *Rattus fuscipes* | Bush rat | Rodentia | 3 | 1.30 | 1.19 | -2.45E-01 | 4.29E-03 |
| *Urocitellus armatus* | Uinta ground squirrel | Rodentia | 6 | 1.13 | 1.11 | -1.73E-02 | 1.68E-03 |
| *Urocitellus armatus* | Uinta ground squirrel | Rodentia | 6 | 1.09 | 1.08 | -1.47E-02 | 1.56E-03 |
| *Urocitellus columbianus* | Columbian ground squirrel | Rodentia | 6 | 1.01 | 0.98 | -3.80E-02 | 3.26E-03 |
| *Urocitellus columbianus* | Columbian ground squirrel | Rodentia | 6 | 1.20 | 1.20 | -3.38E-03 | 6.96E-04 |
| *Ursus americanus subsp. floridanus* | Florida black bear | Carnivora | 4 | 1.02 | 1.02 | -3.68E-03 | 3.97E-04 |
| *Ursus arctos subsp. horribilis* | Grizzly bear | Carnivora | 7 | 1.03 | 1.02 | -1.38E-03 | 1.26E-04 |
| *Ursus maritimus* | Polar bear | Carnivora | 5 | 0.94 | 0.93 | -1.91E-02 | 9.23E-04 |

**Figure S1**. Histogram showing the distribution of sampling effort duration (in years) across the 43 studied mammal populations. The x-axis represents the duration of the study (“Years sampled”).

**Table S2**. The species-specific scales for the elasticity of *λ_1_* to changes in demographic processes and for the self-second derivatives of *λ_1_* with respect to demographic processes for the 16 mammal species studied.

| Figure 3 reference | Species common name | E_max_=elasticity maximum value | SSD_min_=self-second derivative minimum value | SSD_max_=self-second derivative maximum value |
| --- | --- | --- | --- | --- |
| A | Blue monkey | 0.52 | -1.25 | 1.27 |
| B | Columbian ground squirrel | 0.23 | -1.48 | 0.01 |
| C | Eastern chimpanzee | 0.60 | -4.39 | 2.59 |
| D | Human | 0.18 | -0.15 | 0.08 |
| E | Killer whale | 0.55 | -5.72 | 3.43 |
| F | Moose | 0.55 | -0.66 | 0.36 |
| G | Mountain gorilla | 0.81 | -1.46 | 0.28 |
| H | Northern muriqui | 0.72 | -1.17 | 0.35 |
| I | Olive baboon | 0.54 | -0.57 | 1.13 |
| J | Polar bear | 0.26 | -0.73 | 0.54 |
| K | Rhesus macaque | 0.51 | -0.54 | 0.71 |
| L | Root vole | 0.86 | -2.54 | 0.22 |
| M | Soay sheep | 0.56 | -0.22 | 0.40 |
| N | Tammar wallaby | 0.55 | -0.64 | 0.34 |
| O | Verreaux’s sifaka | 0.60 | -2.64 | 1.34 |
| P | White faced capuchin monkey | 0.66 | -2.66 | 1.21 |
